# Exploring a scalable real-time simulation for interprofessional education in pharmacy and medicine

**DOI:** 10.15694/mep.2020.000240.1

**Published:** 2020-10-26

**Authors:** Stephen Duffull, Aynsley Peterson, Bill Chai, Frasier Cho, Justice Opoku, Tasia Sissing, Daniel Smith, Tran Tongskul, Kyle Wilby

**Affiliations:** 1 University of Otago

**Keywords:** interprofessional education, simulation, serious games, distance learning

## Abstract

This article was migrated. The article was marked as recommended.

The organisational logistics and cost of interprofessional education (IPE) remain barriers to widespread embedding of IPE activities in health care training. The ability of students to undertake IPE activities in the form of treating a virtual patient independently of an instructor and facilities would be of potential benefit. In this study we explore the feasibility and potential IPE benefits of SimPHARM, a cloud based simulation platform that allows students to treat virtual patients in a real-time simulation. We enrolled three pairs of medical and pharmacy students and after familiarisation with the platform assigned them a 2 day virtual patient. At the end of the case they completed a questionnaire, an interview and we reviewed their SimPHARM log-files. The results supported the logistical feasibility of this approach. We found that most students interacted asynchronously on the platform, leaving notes for each other, in order to arrive at their clinical decisions. The participants found their interactions meaningful in helping them to make clinical decisions and learn from each other.

## Introduction

Therapeutic decision-making is a fundamental skill that health professionals use in order to make evidence-based and individualised decisions on the choice of and dose selection of medicines to improve patient care ( Wright *et al*., 2019). It differs from clinical reasoning as described in the medical literature which focuses principally on diagnostic reasoning ( Cook *et al*., 2019). Therapeutic decision-making encompasses a range of cognitive skills, such as problem solving, critical thinking, clinical reasoning and judgement, that are used to make therapeutic decisions about drug therapy ( Wright *et al*., 2019). The process of making therapeutic decisions is described by an overarching four step process, information gathering, reasoning, judgement and the enactment of the decision which links with the Pharmacists Patient Care Process. The middle two steps are hidden (generally non-observable to the teacher) and tacit (implied by the outcome but not shown explicitly in process) and are rarely formally approached in health care programmes.

The goal of interprofessional education is the natural formation of a collaborative practice environment in which practitioners from different professions together share in decision-making. Due to the high stakes nature of decision-making for patient care it is desirable to provide an environment for students that is authentic, safe and provides opportunities for reflective learning. Safety here refers to both a safe learning environment, one in which a student can operate with relative freedom without feeling judged by tutors or other students, as well as an environment that does not present a risk to the patient. Typically, simulation environments have been used extensively in IPE. They are however, often prohibitive on resources either due to the need for individual team supervision or the need for specialised simulation equipment. The aim of this initiative is to explore a scalable autonomous IPE opportunity using a real-time simulation.

## Methods

### IPE platform

The tool was a cloud based real-time simulation platform was used (SimPHARM) {see ( Duffull and Peterson, 2020) for further descriptions}. The simulation environment can be accessed by a student from any web browser and continues to run even when the student is not logged on (the system does send alerts though if their virtual patient’s condition worsens). All simulations run in real-time and students cannot pause or rewind a case. Cases can be assigned to individual students (as single player) or teams (as multiple player) and can scale to whole year groups.

### Design and Setting

This exploratory study was conducted at the University of Otago (New Zealand). Participating students were fourth year pharmacy (BPharm) and fourth year medical (MBChB). The fourth year of pharmacy precedes their internship after which they can register as a pharmacist. The fourth year of medicine is followed by a 5 ^th^ year then a trainee intern year following which they can register as a medical practitioner. All students had previously participated in IPE activities and had completed clinical placements, including within hospital settings.

The study was performed in three parts; part 1 involved an initial education session on therapeutic decision-making and SimPHARM training. Part 2; teams of medical and pharmacy students treated a virtual patient. Part 3; participants were invited to attend a debrief session and complete a questionnaire and interview. This study was approved by the University of Otago Human Ethics Committee (D19/170).

**Part 1.** The researchers ran an initial socialisation and education session for participants. The education session aimed to introduce the process of therapeutic decision-making and how to use SimPHARM. The pharmacy students had already experienced the education session and were excluded from this component. During the education session the medical students played as a single player using SimPHARM on a short (1 hour) case (Theo Anderson; Supplementary File 1). Pharmacy and medical students were then asked to form (self-paired) IPE teams. The teams were re-assigned the same case (Theo) to practise working on SimPHARM in a multiplayer setting.

Potential health practitioner roles (e.g. the role of a pharmacist or doctor) were not discussed and no advice was provided about assuming roles during their interaction on SimPHARM. SimPHARM does not limit functionality to roles (i.e. any player can order lab tests and prescribe medicines).

**Part 2.** Each IPE team was assigned a 2-day interactive SimPHARM case (Brian Whitehead, Supplementary File 2). The case involved a polypharmacy case, and the team’s primary goal was management of these medicines to optimise the patient’s outcome.

**Part 3.** An educational debrief session was run one week after participants completed the case. The debrief concentrated on the process of therapeutic decision-making and not on the clinical relevance of any specific decisions. In addition, participants individually completed a purpose-built questionnaire (Supplementary File 3) and participated in an interview. The interview provided an opportunity for participants to discuss their IPE experiences. The interview sessions were not recorded, however notes were taken by the researchers at the interviews.

### Data analysis

Mixed methods were used for data analysis. Data from the questionnaire and SimPHARM log files were summarised using descriptive statistics. Notes taken during the interviews (and any comments from the questionnaires) were transcribed into an electronic format for an inductive thematic analysis. Statements relating to the research questions were coded and subsequently reviewed. Codes were then reviewed to identify themes. Data were specifically reviewed for themes relating to feasibility and engagement.

## Results

Three IPE teams (each comprising 1 pharmacy and 1 medical student) participated in this study. At the time of this study the pharmacy students were currently on campus in class and the medical students were on clinical rotation.

*Questionnaire - quantitative data analysis.* Quantitative data from the survey are reported in Table 1. The data were aggregated into high (response=4 or 5), moderate (response=3) and low (response=2 or 1). It appears from Table 1 that accessing SimPHARM for IPE was feasible (scoring moderate or high in Q1-4). There was a moderate impact on course load (Q5) and decisions were shared with perceptions of learning from each other (Q7-12).

**Table 1.** Responses to Questionnaire

	Questions	Medicine	Pharmacy
1	To what extent were you able to do what you wanted in SimPHARM? (e.g., order lab tests, leave notes etc.)	High	High
2	How many times did you access SimPHARM during your case?	Moderate	High
3	Rate your ability to access SimPHARM (e.g., logging in, internet etc.)	High	High
4	Rate your ease of use of SimPHARM	High	High
5	To what extent did using SimPHARM interfere with your course load	Moderate	Moderate
6	Did interacting with your partner improve your decision making?	High	High
7	Were decisions generally made individually or shared?	Moderate	High
8	Rate your understanding of TD-M/CD-M*	High	Moderate
9	How much do you feel you learned from your partner?	Moderate	High
10	How much did this experience improve your understanding of your future role as a pharmacist/doctor?	Moderate	High
11	Were you able to interact with your partner in a meaningful way?	Moderate	Moderate
12	To what extent were you able to do what you wanted in SimPHARM? (e.g., order lab tests, leave notes etc.)	High	High
13	How often did you interact outside of SimPHARM? (e.g. Facebook, WhatsApp)	Low	Low
14	Would you use SimPHARM again for “IPE”?	High	High

*MPE SimPHARM Survey Responses. The results were translated into low, moderate and high for scale 1-2, 3, and 4-5 respectively. Results from medicine and pharmacy students were aggregated together in the first two columns and the average responses from both medicine and pharmacy students were recorded. CD-M = clinical decision-making; TD-M = therapeutic decision making.*

*SimPHARM-log analysis.* The log files from SimPHARM indicated that the participants regularly left notes for their IPE team partners (two IPE teams left 18 notes). At the time of this study, SimPHARM did not record who wrote the notes so it was generally not possible for the research team to delineate medical from pharmacy notes. Of interest the time stamp between notes suggested the IPE team members were not conferring synchronously. Between five and seven decisions were enacted by each IPE team resulting in de-prescribing, prescribing new medicines or changing the dose of existing medicines.

*Qualitative analysis of survey and interviews.* Three themes were identified from the qualitative data. These were: (1) a grouped theme relating to feasibility, engagement, and immersion, (2) asynchronicity, and (3) meaningful interactions. In addition, a number of comments fell into a “general” category corresponding to overall findings.

### Theme 1: Feasibility, Engagement, and Immersion

Feasibility relates to the functional ability to use SimPHARM. Immersion relates to engagement (use of SimPHARM for care of their virtual patient) and the authenticity of the experiences (i.e. the situation feels real). Feasibility was reflected in the comments *“Easy to navigate and find things”* (M1) and *“Easy to use”* (P2). Engagement and immersion were described *“I got quite attached to him [the patient]” (P3).* A medical student reported that the, “ *simulation was stable and accurate and comprehensive”* (M3). This reinforces the view of the user as seeing SimPHARM as a credible simulation. Furthermore, *“I think it would be very useful for all years of health professional degrees”* (M3), suggesting that realistic scenarios can be created and applicable for a wide range of professional programmes.

### Theme 2: Asynchronicity

Synchronicity relates to the need of IPE team members to be present at the same time/place in order for a decision to be made. No evidence was found to support the need for synchronicity. “ *Half of our communication was over Facebook, and the rest was over SimPHARM*” (P1), showing that further communication was achieved through instant messaging without a need for face to face meetings. Participants commented that the logistics of meeting would be difficult, “ *Scheduling was tricky because we have different timetables”* (P3). Log files from SimPHARM indicated that notes were not left at the same (or close to the same times) by IPE team members

### Theme 3: Meaningful Interaction

Meaningful interaction is used to describe an effective collaboration between partners to improve decision-making. A participant noted that leaving notes allows one to, “ *check your own process more” (*P3). Relating to decision-making, SimPHARM notes were “ *easier to use than charts which are hard to refer [back] to specific decisions*” (M2). Collaborative decision-making assisted individuals when they were, “ *not always confident making decisions by myself”* (P3). Most participants found that interacting with their partners improved their decision-making with comments such as “ *I’m thinking of doing this,” “yeah agree with that”* and *“how about this”.* Participants indicated that they “ *work together more,”* and *“learnt more about what each other does”* (this comment was compared to current IPE programmes at the University of Otago). Also the simulation “uses *each other’s knowledge more”* which was a key factor in learning about how other health professions operate and have their own specialties.

## Discussion

Interprofessional education, learning with, about and from other health professionals is a central part of health care curricular. It is however, exceedingly difficult logistically to organise large groups of students from diverse backgrounds with equally diverse curricular and timetables. In this pilot study we saw that our IPE teams, each comprising 1 pharmacy and 1 medical student, could interact together in a meaningful way to treat their virtual patient. This occurred in their own time and their participation in the game was unsupervised, meaning that upscaling would not impose critical limits on instructors’ time or clinical infrastructure.

Managing time by the students was and remains a critical component for IPE team success. We saw that students were able to interact asynchronously and effectively manage their patient’s needs. This is not dissimilar to the many clinical practice settings where medical and pharmacy practitioners do not consistently co-share clinical space. This study was conducted in addition to each student’s already busy workload. If this was intended to be rolled out into an IPE program then it would be necessary to allow time in the timetable for the IPE commitment but this would not need to be synchronous across all team members.

Upscaling cases for team work is relatively easy in a cloud based environment and since the application runs autonomously then there is no need for instructor time during case running. However, as with all simulation settings the debrief is a critical part of this process and this aspect would remain time critical and could be run online.

It is important to note that this is a pilot study with limited number of teams. We are planning further studies with other health care professional students and larger teams. The results to date appear positive but care is needed when considering the logistics in upscaling particularly around (1) the need for initial socialisation of IPE teams and (2) managing IPE team debriefs.

In conclusion, interprofessional distance education is a practical and feasible way to generate engagement between health professional students.

## Take Home Messages

• Students interacting with a virtual patient can complement current interprofessional education opportunities.
• to complement current interprofessional education opportunities.
• Interprofessional shared decision-making is able to be evaluated on a cloud based simulation platform.
• Pharmacy and Medical students were able to work together and have meaningful interactions as a team even when they were asynchronous in time and location.
• Cloud based simulations can be scaled to large interprofessional cohorts with minimal logistic and infrastructure constraints.
• Further exploration of distance interprofessional education is warranted.

## Notes On Contributors

**Stephen Duffull:** Professor of Pharmacy. Pharmacist. Area of research interest: Pharmacometrics and Education. Supervisor of research team, University of Otago. ORCID ID: https://orcid.org/0000-0002-6545-9408

**Aynsley Peterson:** Professional practice fellow. Pharmacist. Co-supervisor of research team, University of Otago.

**Bill Chai:** Final year undergraduate pharmacy student, member of research team, University of Otago.

**Frasier Cho:** Final year undergraduate pharmacy student, member of research team, University of Otago.

**Justice Opoku:** Final year undergraduate pharmacy student, member of research team, University of Otago.

**Tasia Sissing:** Final year undergraduate pharmacy student, member of research team, University of Otago.

**Daniel Smith:** Final year undergraduate pharmacy student, member of research team, University of Otago.

**Tran Tongskul:** Final year undergraduate pharmacy student, member of research team, University of Otago.

**Kyle Wilby:** Associate Professor of Pharmacy. Pharmacist. Area of research Interest: Education. Co-supervisor of research team, University of Otago. ORCID ID: https://orcid.org/0000-0002-1670-2512

## Declarations

Declaration: SD is the inventor of the game, SimPHARM. No authors have a conflict of interest.

## References

[ref1] Cook D. A. Durning S. J. Sherbino J. and Gruppen L. D. (2019) Management reasoning: implications for health professions educators and research agenda. Academic Medicine. 94(9), pp.1310–1316. 10.1097/ACM.0000000000002768 31460922

[ref2] Duffull S. B. and Peterson A. K. (2020) Student’s perceptions of playing a serious game intended to enhance therapeutic decision-making in a pharmacy curriculum. Currents in Pharmacy Teaching and Learning. [in press] 10.1016/j.cptl.2020.05.011 32867933

[ref3] Wright D. F. B. Anakin M. G. and Duffull S. B. (2019) Clinical decision-making: an essential skill for 21 ^st^ century pharmacy practice. Research in Social and Administrative Pharmacy. 15(5), pp.600–606. 10.1016/j.sapharm.2018.08.001 30100198

