# Supplementary material for: Exploring a scalable real-time simulation for interprofessional education in pharmacy and medicine
Source: MedEdPublish (2016). 2020 Oct 26;9:240. [Version 1] doi: 10.15694/mep.2020.000240.1 (PMC10697448; doi:10.15694/mep.2020.000240.1)
Supplement: Peer review history

# Reviewer report: Reviewer response for version 1

**DOI:** 10.21956/mep.20184.r31215

**Published:** 2021-01-17

**Authors:** Ken Masters

**Affiliations:** 1 Sultan Qaboos University

This review has been migrated. The reviewer awarded 4 stars out of 5 A really interesting case study dealing with simulated patients in an IPE setting with medical and pharmacy students.Yes, IPE is a wonderful concept; as the authors note, though, the logistics of implementation are daunting, and, if a simulation can help to overcome the logistical problems, then a great deal will have been achieved.Although this is a small Pilot study, it has been well-conducted, and the results indicate that a larger rollout would be justified. I particularly liked the fact that the researchers did not rely only on the student feedback, but also analysed the logs. It is a pity that the system does not give more detailed identifiers, as this would have allowed for a more comprehensive data analysis. Nevertheless, from the available data, the researchers have been able to draw useful conclusions.Just one thing: when using any technology, there is a cost/benefit to be considered. If contractual agreements allow, it would be useful if the authors could give some idea of the systems’ costs, student computing minimum requirements, etc.I look forward to the next paper that will explore this concept with a larger group of students.

Reviewer Expertise:

NA

No decision status is available

# Reviewer report: Reviewer response for version 1

**DOI:** 10.21956/mep.20184.r31217

**Published:** 2020-11-03

**Authors:** Hui Meng Er

**Affiliations:** 1 International Medical University

This review has been migrated. The reviewer awarded 3 stars out of 5 This is an interesting pilot study that aims to explore the potential of a simulation platform to foster interprofessional education (IPE) among pharmacy and medical students. Logistic constraint has always been a barrier to the implementation of IPE. With the use of a simulation platform where the students can log in at their own time, the students were able to interact with each other asynchronously in managing the virtual patient and learn from the experience together. It would have been good if more details about the IPE simulation platform is provided, for example, whether the response of the virtual patient is dependent on the student’s decision, and whether prompt feedback is given if the incorrect decision is made (though it is stated that alerts are sent if the virtual patient’s condition worsens). In Part 3, the debrief session focusses on the therapeutic decision making instead of clinical relevance. While the earlier could be the main competency addressed, both are equally important and should be addressed simultaneously in a simulation environment especially for students in senior years. Perhaps these areas can be further explored beyond the pilot study.

Reviewer Expertise:

NA

No decision status is available

# Reviewer report: Reviewer response for version 1

**DOI:** 10.21956/mep.20184.r31216

**Published:** 2020-10-26

**Authors:** Shamaila ijaz

**Affiliations:** 1 Lisca

This review has been migrated. The reviewer awarded 4 stars out of 5 The abstract is summarized nicely. The Introduction relates the data. I couldn't find the sample size in the study. The results are interesting. Then discussion focus on its uses, but other references should have been added. Lets see what results are found while the study is done with a increase in sample size and whether it can be generalized and to what extent.

Reviewer Expertise:

NA

No decision status is available
